# The Principles of Physiology Applied to the Preservation of Health, and to the Improvement of Physical and Mental Education

**Published:** 1835-04-01

**Authors:** 


*The Principles of Physiology applied to the Preservation of Health, and to the Improvement of Physical and Mental Education.* By ANDREW COMBE, M.D., Fellow of the Royal College of Physicians of Edinburgh. *Third Edition, revised and enlarged.*—Edinburgh, 1835. Post 8vo. pp. 404.

WE are glad to find that the favourable opinion which we gave of the first edition of this work, in our fourth Number, has been confirmed by critics and readers; and that a third edition has, like its predecessor, afforded Dr. Combe the opportunity of adding and correcting. The book is written in the plain and unpretending style which befits a man who is discussing important subjects with earnestness and sincerity. The following extracts consist in part of the matter which has been added since the original publication of this useful compendium.

"I have often had occasion to remark the powerful influence which free perspiration from natural causes has in relieving acidity in the stomach and promoting digestion, and the fact that acidity is most prevalent when the skin is most inactive; and have thereby been led to prescribe with advantage the frequent use of the tepid and vapour bath in calculous and other complaints arising from excess of acid. In accordance with the same principle, we find Lord Byron noting in his journal (28th March, 1814), that after having, when previously very unwell, 'sparred with Jackson *ad sudorem*,' he felt 'much better in health than for many days,' and remarking, that 'the more violent the fatigue, *the better his spirits for the rest of the day*,' and this too at a time when he was deriving little relief from his favourite remedies, abstinence and soda water.

"These results seem to corroborate the doctrine of M. Donné, that in the healthy state an acid humour is secreted from the whole surface of the skin, while the mucus secreted from the digestive canal is everywhere, except in the stomach, of an alkaline nature. The subject is still not very clear, but it deserves the most careful examination." (P. 108.)

"So little, indeed, are we taught to think of the nature and wants of the human constitution, that in Edinburgh, and almost every large town, we have instances of large public rooms, capable of holding from 800 to 1000 persons, built within these few years, without any means of adequate ventilation being provided, and apparently without the subject having ever cost the architect a thought! When these rooms are crowded and the meeting lasts for some hours, especially if it be in winter, the consequences are sufficiently marked. Either such a multitude must be subjected to all the evils of a contaminated and unwholesome atmosphere, or they must be partially relieved by opening the windows, and allowing a continual stream of cold air to pour down upon the heated bodies of those who are near them, till the latter are thoroughly chilled, and perhaps fatal illness is induced; and unfortunately, even at such a price, the relief is only partial: for the windows being all on one side of the room, and not extending much above half-way to the ceiling, complete ventilation is impracticable. This neglect is glaringly the result of ignorance, and could never have happened, had either the architects or their employers known any thing of the laws of the human constitution; and yet it is still doubted whether it be prudent or right to teach the intelligent portion of the community any knowledge of the structure and uses of their own organs!

"These remarks have been fully verified since they were first printed. During the last winter an unusual number of courses of popular lectures were given in Edinburgh, many of which were very fully attended. From the utter impossibility of safe ventilation, those courses which were most crowded, were accessible only at such an expense of health and suffering on the part of their less robust auditors, as served to neutralize, in a great measure, the advantages which might otherwise have been derived from them. Several of my own friends were compelled to discontinue their attendance, while others persevered, although at the certain cost of a severe headach. This nuisance is the more to be regretted, as it has arisen solely from the architects and the public not having been sufficiently alive to the importance of procuring that prime necessary of life, pure air; and not at all from any difficulty of obtaining it, which could not at the first have been easily overcome." (P. 230.)

